# Resting‐State Activity and Connectivity of Dopaminergic Key Areas and Outcome After a Severe Stroke

**DOI:** 10.1111/ejn.70507

**Published:** 2026-04-15

**Authors:** Hanna Braaß, Liv Asmussen, Winifried Backhaus, Focko L. Higgen, Philipp J. Koch, Pawel Wrobel, Chi‐un Choe, Fanny Quandt, Benedikt Frey, Robert Schulz

**Affiliations:** ^1^ Department of Neurology University Medical Center Hamburg‐Eppendorf Hamburg Germany; ^2^ Department of Systems Neuroscience University Medical Center Hamburg‐Eppendorf Hamburg Germany; ^3^ Department of Neurology University of Lübeck Lübeck Germany

**Keywords:** ALFF, brain reserve, dopamine, fMRI, functional connectivity

## Abstract

Brain reserve capacity has recently gained an increasing interest in stroke recovery research to provide a deeper understanding of outcome variability. For instance, global and focal parameters of brain health, such as white matter hyperintensity burden or the structural reserve of the cerebellum, have been linked to recovery. Recently, it was shown that the pre‐stroke structural state of key areas of the dopaminergic network might influence outcomes after stroke. We reanalyzed resting‐state functional MRI data of 19 severely impaired acute stroke patients and 19 healthy controls and computed amplitudes of low‐frequency fluctuations (ALFF) and functional connectivity (FC) in and between eight subcortical and cortical areas of the nigrostriatal and mesocorticolimbic dopaminergic network of the contralesional hemisphere. Linear regression modeling was used to compare patients and controls and combine patients' ALFF and FC data with clinical follow‐up data obtained after 3–6 months. The group comparison revealed a significant upregulation of ALFF in the prefrontal cortices, the ventral tegmental area, the nucleus accumbens, and the caudate nucleus. Additionally, for some regions and connections within the nigrostriatal and mesocorticolimbic network, ALFF and FC estimates were significantly linked to global disability and symptom burden at follow‐up. These data indicate a link between the pre‐stroke functional state of key areas and pathways of the contralesional dopaminergic system and recovery from a severe stroke, thereby adding novel functional insights to recent structural data and promoting the emerging concepts of brain reserve capacity after stroke.

## Graphical abstract

Our study examined whether functional properties of the contralesional dopaminergic system relate to recovery after severe stroke. Resting‐state fMRI from 19 acute stroke patients and 19 controls was used to quantify ALFF and functional connectivity across nigrostriatal and mesocorticolimbic regions. Patients showed increased ALFF in the prefrontal cortex, the ventral tegmental area, the nucleus accumbens, and the caudate. ALFF and connectivity metrics predicted disability and symptom burden at 3–6 months, supporting a functional dopaminergic brain reserve contribution to stroke recovery.

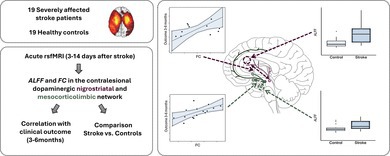

**Abbreviations**
ALFF: amplitudes of low‐frequency fluctuations
AMYG: amygdala
CAU: caudate nucleus
DWM: deep white matter
FC: functional connectivity
HIP: hippocampus
MRS: Modified Rankin Scale
NACC: nucleus accumbens
NIHSS: National Institutes of Health Stroke Scale
PFC: medial prefrontal cortex
PUT: putamen
PVWM: periventricular white matter
ROI: region of interest
SN: substantia nigra (pars compacta)
VTA: ventral tegmental area

## 1 Introduction

Brain reserve capacity (Umarova et al. 2024) has recently gained an increasing interest in stroke recovery research to better understand outcome variability. For instance, global and focal parameters of brain health, such as white matter hyperintensity burden or the structural reserve of the cerebellum and contralesional motor areas (Hong et al. 2021; Rojas Albert et al. 2022; Sadeghihassanabadi et al. 2022), have been linked to recovery. Although previous analyses were mainly focused on motor brain networks, we have recently investigated the structural state of key areas of a distinct network defined by dopamine as the leading neurotransmitter (Asmussen et al. 2024). Comprising nigrostriatal and mesocorticolimbic pathways (Bjorklund and Dunnett 2007) connecting the substantia nigra with the caudate nucleus and putamen on the one hand and the ventral tegmental area with the amygdala, the nucleus accumbens, the hippocampus, and the medial prefrontal cortex (PFC) on the other hand, this dopamine network critically contributes to various motor and cognitive functions, such as planning and execution of voluntary movements (Sawada et al. 2015), learning (Wachter et al. 2009; Hosp et al. 2011), motivation (Bromberg‐Martin et al. 2010; Myers et al. 2016), reward (Lammel et al. 2014; Ruiz‐Tejada et al. 2022), memory processes (Abe et al. 2011), and reward‐driven motor learning (Widmer et al. 2019; Vassiliadis et al. 2021). It has been hypothesized that these processes are critically involved in relearning lost functions, acquiring novel skills, and engaging and adhering to neurorehabilitation (Krakauer 2006; Dipietro et al. 2012; Gangwani et al. 2022; Widmer et al. 2022).

We found that larger volumes of the amygdala and the nucleus accumbens at baseline were positively associated with a more favorable outcome. This suggested a link between the structural state of mesolimbic areas contributing to motor learning, motivational and reward‐related brain networks, and potentially the success of neurorehabilitation (Asmussen et al. 2024). It added a reserve perspective for dopaminergic brain networks to previous studies reporting that basal ganglia lesions (Fries et al. 1993; Boyd and Winstein 2004), nigrostriatal white matter tract damage (Rimmele et al. 2018), or lesions affecting the nucleus accumbens (Skidmore et al. 2022) were linked to worse recovery and reduced treatment gains during specific training paradigms, respectively. Animal stroke models (Obi et al. 2018) and findings in traumatic brain injury (Chen et al. 2017; Jenkins et al. 2018) have suggested that dopamine might be a promotor of recovery, and dopamine levels might be linked to reductions in oxidative stress and improvement in neuronal survival.

Building on our previous work on the importance of structural dopaminergic network parameters for stroke recovery (Asmussen et al. 2024), the present study questioned to what extent the functional state of key areas and interregional coupling of contralesional dopaminergic networks might inform about recovery after stroke. To answer this question, we reanalyzed resting‐state functional MRI data of 19 severely impaired acute stroke patients (Backhaus et al. 2021) acquired 3–14 days after the event. Nineteen healthy controls of similar age and gender were also included in the study. We computed amplitudes of low‐frequency fluctuations (ALFF), a surrogate of regional spontaneous brain activity, and functional connectivity (FC) in and between eight subcortical and cortical key areas of the contralesional dopaminergic nigrostriatal and mesocorticolimbic networks. Linear regression modeling was used to compare stroke patients and controls and integrate patients' ALFF and FC data with clinical follow‐up data obtained after 3–6 months. We hypothesized to find significant positive associations between regional spontaneous activity and FC and outcome after stroke.

## 2 Participants and Methods

### 2.1 Cohort and Clinical Data

This study is based on published clinical and imaging data of 19 more severely impaired acute stroke patients admitted to the University Medical Center Hamburg‐Eppendorf from October 2017 to February 2020 (Backhaus et al. 2021). The original study was approved by the Medical Association of Hamburg (PV5442). Acute stroke patients (3–14 days after the incident) were included according to the following criteria: first‐ever ischemic stroke causing a severe motor deficit involving hand function, Modified Rankin Scale (MRS) > 3 or Barthel index ≤ 30 or “early rehabilitation” Barthel index < 30 and age ≥ 18 years. Exclusion criteria were preexisting clinically silent brain lesions of > 1 cm^3^ or preexisting motor deficits, contraindications for MRI, relevant psychiatric diseases, drug abuse, or pregnancy. The original report includes a flowchart of patients' inclusion. All participants provided informed consent themselves or via a legal guardian, following the ethical Declaration of Helsinki. Acute stroke patients underwent structural and functional resting‐state MRI within the first days after the event as time point T1 (Days 3–14). Clinical follow‐up time point T2 was defined in the late subacute stage of recovery (Bernhardt et al. 2017) after 3 months or in patients where clinical data for this time point were unavailable after 6 months. The initial symptom burden at time point T1 was quantified using the National Institutes of Health Stroke Scale (NIHSS), and the outcome at time point T2 was operationalized by the MRS and the NIHSS. For group comparisons, 19 healthy control participants without preexisting neurological or psychiatric conditions, matched to patients by age and sex, were included and analyzed. They were recruited from the participant's database of the lab. All patients and controls were right‐handed.

### 2.2 Brain Imaging: Data Acquisition

A 3‐T Skyra MRI scanner (Siemens Healthineers, Erlangen, Germany) equipped with a 32‐channel head coil was used to acquire multimodal imaging data, including structural high‐resolution T1‐weighted and functional resting‐state images. For the T1‐weighted sequence, a three‐dimensional magnetization‐prepared rapid gradient echo (3D‐MPRAGE) sequence was used with the following parameters: repetition time (TR) = 2500 ms, echo time (TE) = 2.12 ms, flip angle 9°, 256 coronal slices with a voxel size of 0.8 × 0.8 × 0.9 mm^3^, field of view (FOV) = 240 mm. The resting‐state functional MRI parameters were as follows: FOV = 260 mm, TR = 2 s, TE = 30 ms, a 72 × 72 × 32 matrix, voxel size 3 × 3 × 3 mm^3^, flip angle 90°, and 210 images. Before the resting‐state scans, the participants were asked to focus on a black cross on a screen. All resting‐state and T1‐weighted images with right‐sided stroke lesions were flipped to the left hemisphere for analysis. This hemispheric flip (T1‐weighted and fMRI images) was also performed in the controls matched to the patients with right‐sided stroke lesions to account for the distribution of stroke lesions to the dominant and nondominant hemispheres. This procedure was in line with the original report (Backhaus et al. 2021). For the T2‐weighted images, a fluid‐attenuated inversion recovery sequence was used with the following parameters: TR = 9000 ms, TE = 86 ms, TI = 2500 ms, flip angle 150°, 43 transversal slices with a voxel size of 0.7 × 0.7 × 3.0 mm^3^, FOV = 230 mm.

### 2.3 Brain Imaging: Image Analysis

The resting‐state and T1‐weighted images were preprocessed using the CONN toolbox v22.a (Whitfield‐gabrieli and Nieto‐castanon 2012), an SPM12‐based toolbox. The default pipeline for volume‐based analyses with the following steps and parameters was used for image preprocessing: The first 10 volumes were discarded to account for magnetization equilibrium effects. During the initial pre‐processing, all functional images were realigned (motion corrected), centered, slice time corrected, and corrected for motion artifacts using the artifact detection tools (ART). All structural and functional images were spatially normalized to MNI space, and the functional images were spatially smoothed to allow for better registration and reduction of noise using a 6 mm full width at half maximum (FWHM) Gaussian kernel (Martino et al. 2018). Functional and anatomical data were segmented into gray matter, white matter, and cerebrospinal fluid (CSF) tissue classes using SPM12 unified segmentation and normalization procedure. After normalization, every image was visually checked for possible registration errors due to the large stroke lesions. After preprocessing, motion parameters were derived from rigid‐body realignment and their derivatives. Five potential noise components (average blood oxygenation level‐dependent [BOLD] signal and the first four components in principal component analysis of the covariance within the subspace orthogonal to the average BOLD signal) derived from CSF and white matter using the aCompCor (anatomical component‐based noise correction) procedure were regressed from the signal (Behzadi et al. 2007) to reduce the confounding effect of physiological fluctuations. The analysis did not include global signal regression to avoid false anti‐correlations (Murphy et al. 2009). A temporal band‐pass filter between 0.008 and 0.1 Hz was applied to focus on slow‐frequency fluctuations while minimizing the influence of physiological, head motion, and other noise sources (Hallquist et al. 2013). The Fazekas score for periventricular white matter (PVWM) and deep white matter lesions (DWM) (Fazekas et al. 1987) was determined using the T2‐weighted images, if available.

### 2.4 First‐Level ALFF Analysis

**FIGURE 1:**
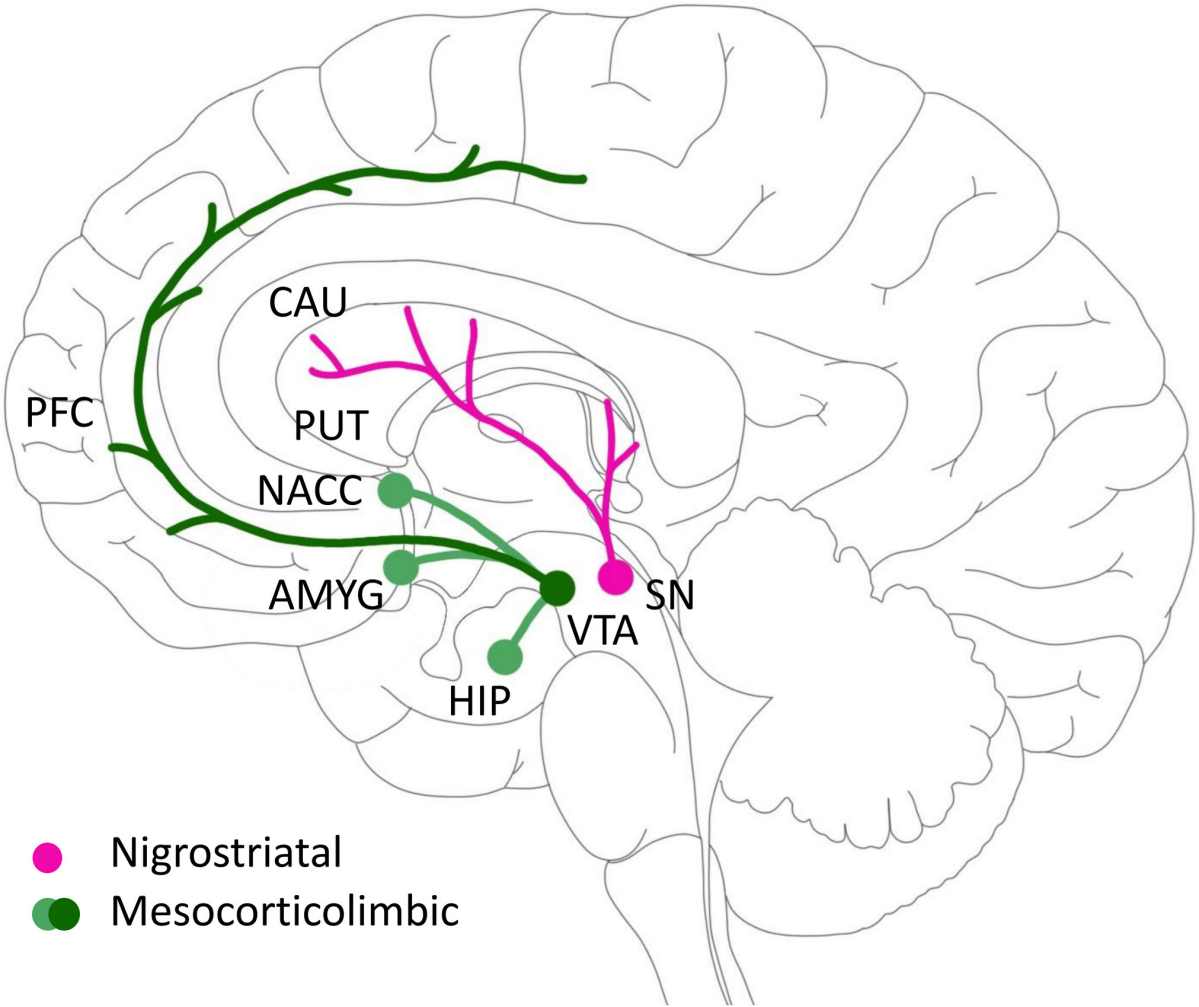
Dopaminergic pathways. This illustration gives an overview of the main dopaminergic pathways. Dopaminergic neurotransmission derives mainly from the substantia nigra (pars compacta, SN) and the ventral tegmental area (VTA) but also from the hypothalamus. Neurons from the substantia nigra project to the dorsal striatum, including the putamen (PUT) and the caudate nucleus (CAU), whereas neurotransmission from VTA targets limbic structures, such as the amygdala (AMYG), the hippocampus (HIP), the nucleus accumbens (NACC), and the medial prefrontal cortex (PFC). Adapted from Asmussen et al. (2024).

The ALFF maps characterizing low‐frequency BOLD signal variability at each voxel was estimated as the root mean square (RMS) of the BOLD signal after denoising and band‐pass filtering between 0.008 and 0.1 Hz. The mean ALFF values of each ROI for each subject were extracted to characterize the spontaneous brain activity of the ROIs. In line with our previous study (Asmussen et al. 2024) on structural brain reserve in key regions of the dopaminergic brain networks, the following eight ROIs were chosen from the AAL3 atlas (Rolls et al. 2020) in the right contralesional hemisphere according to the composition of the nigrostriatal and mesocorticolimbic networks (Figure 1): substantia nigra (pars compacta, SN), putamen (PUT), caudate nucleus (CAU), ventral tegmental area (VTA), nucleus accumbens (NACC), amygdala (AMYG), and hippocampus (HIP). For medial PFC, ALFF and FC values were averaged for the following labels: superior frontal gyrus, medial orbital part, superior frontal gyrus, medial part, and middle frontal gyrus.

### 2.5 First‐Level FC Analysis (ROI‐to‐ROI Analysis)

In addition to extracting the mean ALFF of each ROI, an ROI‐to‐ROI analysis was performed. For the ROI‐to‐ROI analysis, the Fisher‐transformed bivariate correlation coefficients (FC values) between each pair of ROI‐BOLD signals were calculated and used for further analysis (Backhaus et al. 2021; Braass et al. 2023).

### 2.6 Statistical Analysis

Statistical analyses were performed in R (Version 4.0.4). For group comparisons, we used linear regression models. Estimated mean values with 95% confidence intervals (CI) are given for stroke patients and healthy participants. Statistical significance was computed using an analysis of variance (ANOVA). For ALFF‐ and FC‐outcome correlations, linear multiple regression models were calculated with MRS or NIHSS obtained at T2 as the dependent variables of interest. Region‐specific ALFF and connection‐specific FC values (from time point T1) were the factors of interest. Models were adjusted for lesion volume (log‐transformed), and NIHSS was obtained at T1. Factor coefficients, including 95% CI, are given. The statistical significance of model improvement was computed using likelihood‐ratio (LR) testing of models without and with the factors of interest. The Akaike Information Criterion (AIC) change was additionally explored to assess model improvement (ΔAIC). The level of significance was set to *P*_FDR_ < 0.05, corrected for multiple comparisons (*n* = 14 for group comparisons, *n* = 28 across MRS/NIHSS correlations) separately for group comparisons, and MRS and NIHSS correlations using the false discovery rate (FDR) method.

## 3 Results

### 3.1 Demographic and Clinical Data

**FIGURE 2:**
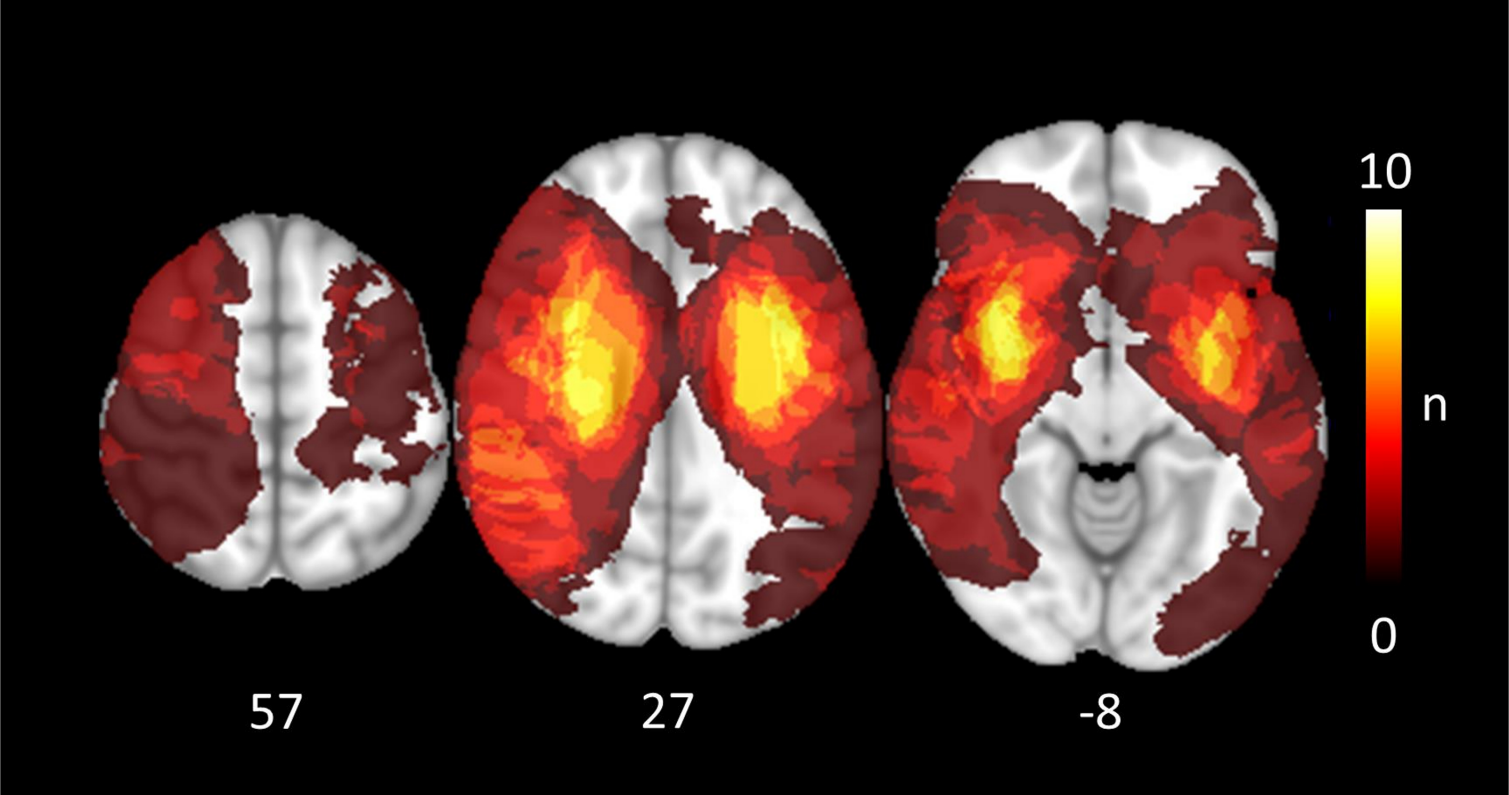
Frequency map of the stroke lesions in the right and left hemispheres overlaying a T1‐weighted template in MNI space (z‐coordinates below each slice). The color intensity indicates the number of subjects of whom lesion voxels lay within the colored region.

Table 1 summarizes the demographic and clinical data of the 19 patients. For group comparison, 19 healthy controls of similar age and gender were also analyzed. The groups did not differ in age (in years, mean ± standard deviation; stroke: 75.3 ± 7.5, control: 73.8 ± 5.8, *p* = 0.50) nor in sex distribution (12 female, seven male in both groups). Figure 2 depicts a lesion map.

**TABLE 1:** Demographic and clinical data.

ID	Age	Sex	History	Side, Dom	TPA/MT (TICI)	LVO	LV	NIHSS T1	MRS T2	NIHSS T2	PVWM/DWM
1	78	f	HT, HC	Left/d	No/no	None	33.6	10	3	3	2/2
2	63	m	HT, DM, HC	Left/d	No/no	M1	55.8	13	1	1	1/1
3	73	f	HT, AF, HThy	Left/d	Yes/yes (2B)	M1	14.4	9	3	3	—
4	73	f	—	Right/n	Yes/yes (2A)	M1	27.6	5	1	2	1/1
5	79	f	MG	Right/n	Yes/yes (2B)	M2/A1	120.4	8	4	2*	2/2
6	89	f	HT	Right/n	Yes/no	None	2.6	7	3	3	2/2
7	71	f	HT, AF	Right/n	Yes/yes (2A)	ACI/M1/A1	38.4	9	3*	—	—
8	76	m	—	Right/n	Yes/yes (3)	M1/A2	101	11	3	—	—
9	78	m	HT, DM, AF	Right/n	Yes/yes (3)	M2	178.1	17	4	3	1/1
10	85	f	HT, DM, AF, BAVR, HC	Right/n	No/yes (2B)	M1	33.5	15	5	14	—
11	78	m	HT, AF	Left/d	No/no	M1	58.1	17	5*	—	2/2
12	74	m	HT, DM, HC, AF	Left/d	Yes/yes (2B)	M1	303.3	24	5	—	—
13	69	m	HT, HUR	Left/d	Yes/yes (2A)	M1	98.4	18	—	—	—
14	77	f	HT, Asthma, AF	Right/n	Yes/no	M1	286.7	11	4*	10*	2/2
15	67	f	OS	Right/n	Yes/no	None	7.4	11	3	7	3/2
16	58	f	HT, HC	Left/d	No/no	ACI/M1	58.4	23	—	—	—
17	80	f	HT, AF	Left/d	No/no	M1	20.5	11	4*	15*	3/2
18	83	f	HT, AF, CAD	Left/d	Yes/yes (3)	Carotis‐T	21.6	10	—	—	3/2
19	80	m	HT, DM, HC, AF, OBE	Right/n	Yes/yes (0)	M1	108.4	16	6	—	—
Pat	75.3 ± 7.5	7 m									2 ± 1/2 ± 0.5
Cont	73.8 ± 5.8	7 m									2 ± 1/2 ± 0.5

*Note:* T1 NIHSS score at the time of study inclusion (time point T1). T2 follow‐up time point after 3 or 6 (indicated by asterisks) months. PVWM (periventricular white matter) and DWM (deep white matter) Fazekas score (Fazekas et al. 1987) (in the control group, only 12 participants received T2 weighted structural MRI images).

Abbreviations: AF, atrial fibrillation; BAVR, biological aortic valve replacement; CAD, coronary artery disease; Cont, controls; DM, diabetes mellitus; Dom, hemispherical dominance with d (dominant hemisphere affected), n (non‐dominant hemisphere affected); f, female; HC, hypercholesterinemia; HT, hypertension; HThy, hyperthyroidism; HUR, hyperuricemia; LV, lesion volume (mL); LVO, large vessel occlusion with occluded vessels; m, male; MG, monoclonal gammopathy; MT, mechanical thrombectomy with TICI (thrombolysis in cerebral infarction grading system: partial perfusion of the treated vessel is reached in Grade 2B); OBE, obesity; OS, osteoporosis; Pat, patients; Side, lesion side; TPA, thrombolysis.

### 3.2 Group Differences Between Stroke and Controls

**FIGURE 3:**
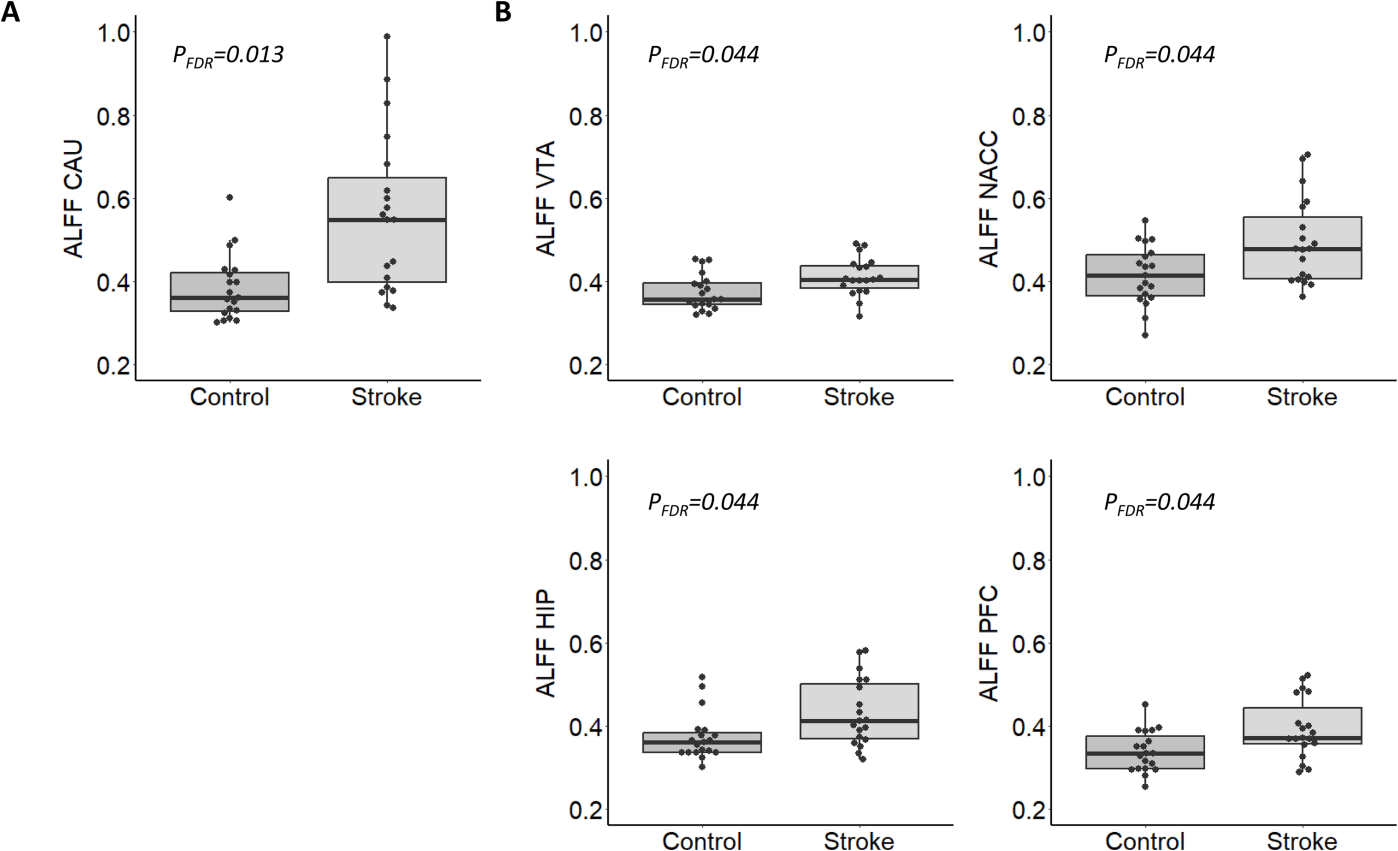
Graphical representation of the ALFF values of both groups. Only the regions with significant group differences from Table 2 are shown. (A) Nigrostriatal regions. (B) Mesocorticolimbic regions.

Stroke patients exhibited a significant upregulation in spontaneous regional brain activity (ALFF) in key areas of the contralesional nigrostriatal and mesocorticolimbic network in the caudate nucleus (CAU), the ventral tegmental area (VTA), the nucleus accumbens (NACC), the hippocampus (HIP), and the PFC. FC group differences were not evident (Table 2 and Figure 3).

**TABLE 2:** Estimated mean values of region‐specific ALFF and coupling‐specific FC with 95% confidence intervals (CI, lower, upper boundary) for stroke patients (mean stroke) and controls (mean control). Statistical significance *P* for the main effect group is derived from ANOVA. *P*_FDR_ values are given corrected for 14 tests. Asterisks highlight significant group differences.

Method	Network	Region	Mean stroke (95% CI)	Mean control (95% CI)	*P*_FDR_
ALFF	Nigrostriatal	SN	0.37 (0.35, 0.39)	0.35 (0.33, 0.38)	0.554
		PUT	0.3 (0.28, 0.32)	0.29 (0.27, 0.3)	0.474
		CAU	0.56 (0.49, 0.63)	0.39 (0.32, 0.45)	0.013*
	Mesocorticolimbic	VTA	0.41 (0.39, 0.43)	0.37 (0.35, 0.39)	0.044*
		NACC	0.49 (0.45, 0.53)	0.42 (0.37, 0.46)	0.044*
		AMYG	0.44 (0.4, 0.48)	0.4 (0.37, 0.44)	0.355
		HIP	0.43 (0.4, 0.46)	0.37 (0.34, 0.41)	0.044*
		PFC	0.39 (0.36, 0.42)	0.34 (0.31, 0.37)	0.044*
FC	Nigrostriatal	SN–CAU	0 (−0.06, 0.06)	−0.03 (−0.09, 0.04)	0.595
		SN–PUT	0.06 (−0.02, 0.14)	0.08 (0, 0.16)	0.718
	Mesocorticolimbic	VTA–NACC	−0.05 (−0.11, 0.01)	0.02 (−0.03, 0.08)	0.154
		VTA–AMYG	0.09 (0.01, 0.17)	0.04 (−0.04, 0.13)	0.554
		VTA–HIP	0.12 (0.05, 0.19)	0.07 (−0.01, 0.14)	0.474
		VTA–PFC	0.01 (−0.04, 0.06)	−0.01 (−0.06, 0.04)	0.629

### 3.3 Outcome Correlations

**FIGURE 4:**
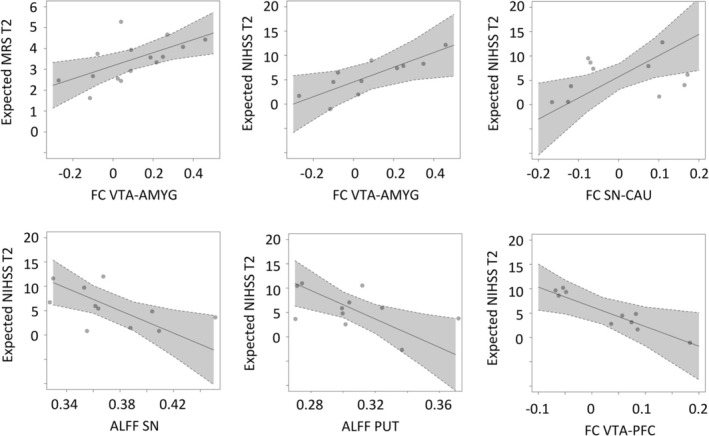
Significant correlations between region‐specific ALFF and connection‐specific FC values and outcomes after stroke. Adjusted effects plots are given for linear regressions with linear fit (gray line), 95% confidence intervals (shaded), and individual point estimates.

Tables 3 and 4 summarize the results of the correlation analyses. Figure 4 illustrates significant ALFF/FC results. For MRS, we found a positive correlation between stronger functional coupling between VTA and the amygdala and more persistent disability at follow‐up (*P*_FDR_ = 0.021, ΔAIC = −6.72). For NIHSS, we observed a positive correlation between higher FC of VTA–AMYG and more symptom burden (*P*_FDR_ = 0.028, ΔAIC = −5.63). SN–CAU coupling also correlated with more deficits at follow‐up (*P*_FDR_ = 0.021, ΔAIC = −7.03). In contrast, VTA–PFC coupling was negatively related to NIHSS at T2, indicating that patients with higher coupling exhibited lower NIHSS scores (*P*_FDR_ = 0.022, ΔAIC = −6.33). Finally, ALFF in SN (*P*_FDR_ = 0.021, ΔAIC = −7.63) and PUT (*P*_FDR_ = 0.021, ΔAIC = −7.83) negatively correlated with NIHSS at T2. For NIHSS, the same findings were evident when using ΔNIHSS as the dependent variable instead of NIHSS at T2 (data not shown). Importantly, none of the 14 ALFF/FC values improved models to explain variance in the initial deficit as operationalized by NIHSS at T1 (all *P*_FDR_ > 0.22), which underlines the direct effects of ALFF/FC onto recovery trajectories instead of the initial deficit.

**TABLE 3:** Estimated coefficients with 95% confidence intervals (CI, lower, upper boundary) for linear relationships between region‐specific ALFF and coupling‐specific FC values and MRS after 3–6 months after stroke (*N* = 16). Results are corrected for lesion volume and NIHSS at time point T1. Statistical significance *P*_FDR_ is derived from LR‐based model comparison (models without and with the ALFF/FC value, corrected for 28 tests across MRS and NIHSS models). Change in Akaike Information Criterion (AIC) is given. Asterisks highlight significant associations.

Method	Network	Region/coupling	Coef. (95% CI)	*P*_FDR_	ΔAIC
ALFF	Nigrostriatal	SN	−11.54 (−23.4, 0.32)	0.096	−3.12
		PUT	−10.86 (−27.92, 6.2)	0.378	−0.42
		CAU	0.96 (−3.08, 5.01)	0.903	1.68
	Mesocorticolimbic	VTA	−0.04 (−13.73, 13.64)	0.994	1.98
		NACC	1.67 (−4.68, 8.01)	0.903	1.58
		AMYG	−1.06 (−9.67, 7.54)	0.920	1.88
		HIP	−1.07 (−9.21, 7.08)	0.920	1.88
		PFC	0.74 (−8.49, 9.97)	0.940	1.98
FC	Nigrostriatal	SN–CAU	1.34 (−5.44, 8.11)	0.903	1.78
		SN–PUT	0.37 (−3.69, 4.43)	0.940	1.98
	Mesocorticolimbic	VTA–NACC	3.2 (−2, 8.4)	0.378	−0.22
		VTA–AMYG	3.13 (0.82, 5.44)	0.021*	−6.72
		VTA–HIP	2.52 (−1.16, 6.21)	0.347	−0.72
		VTA–PFC	−0.95 (−6.85, 4.94)	0.912	1.88

Abbreviation: CI confidence interval.

**TABLE 4:** Estimated coefficients with 95% confidence intervals (CI, lower, upper boundary) for linear relationships between region‐specific ALFF and coupling‐specific FC values and NIHSS after 3–6 months after stroke (*N* = 11). Results are corrected for lesion volume and NIHSS at time point T1. Statistical significance *P*_FDR_ is derived from LR‐based model comparison (models without and with the ALFF/FC value, corrected for 28 tests across MRS and NIHSS models). Change in Akaike Information Criterion (AIC) is given. Asterisks highlight significant associations.

Method	Network	Region/coupling	Coef. (95% CI)	*P*_FDR_	ΔAIC
ALFF	Nigrostriatal	SN	−115.37 (−202.41, −28.34)	0.021*	−7.63
		PUT	−146.19 (−254.9, −37.49)	0.021*	−7.83
		CAU	−0.66 (−23.91, 22.59)	0.994	1.97
	Mesocorticolimbic	VTA	42.98 (−75.04, 161)	0.746	0.87
		NACC	0.69 (−48.86, 50.25)	0.994	1.97
		AMYG	−13.55 (−78.53, 51.42)	0.903	1.67
		HIP	−12.92 (−76.63, 50.79)	0.903	1.67
		PFC	−18.12 (−78.71, 42.47)	0.896	1.27
FC	Nigrostriatal	SN–CAU	43.55 (8.96, 78.14)	0.021*	−7.03
		SN–PUT	−3.88 (−28.7, 20.94)	0.903	1.77
	Mesocorticolimbic	VTA–NACC	−6.63 (−44.93, 31.66)	0.903	1.77
		VTA–AMYG	15.12 (1.61, 28.62)	0.028*	−5.63
		VTA–HIP	5.4 (−21.22, 32.01)	0.903	1.67
		VTA–PFC	−40.47 (−74.44, −6.49)	0.022*	−6.33

Abbreviation: CI confidence interval.

When integrating group comparison (Table 2) and correlation outcome data (Tables 3 and 4), we observed that particularly those regions and couplings exhibiting significant associations with the outcomes after stroke did not show significant differences compared with healthy controls, indicating that they might be interpreted as estimates of premorbid characteristics of dopaminergic brain networks influencing recovery after stroke: Patients with stronger ALFF activity in nigrostriatal areas, such as the SN or PUT, or stronger mesocorticolimbic FC between VTA and the PFC, might be at better odds of a more favorable outcome after stroke. In contrast, enhanced FC between the VTA and the AMYG and between the SN and CAU were linked to a worse outcome. Regions and couplings that showed significant upregulations in regional ALFF and interregional FC did not correlate with the outcome after stroke.

## 4 Discussion

The main finding of this study was that stroke patients exhibited significant upregulations of spontaneous regional brain activity in key areas of the nigrostriatal and mesocorticolimbic dopaminergic network of the contralesional hemisphere. Additionally, for some regions and connections that did not show stroke‐related alterations, baseline activity and coupling estimates were significantly linked to global disability and symptom burden after 3–6 months. The present data add novel functional insights into the importance of dopaminergic brain networks after stroke, thereby promoting the emerging concepts of brain reserve to better understand outcome variability.

Dopaminergic nigrostriatal and mesocorticolimbic networks have gained an increasing interest in stroke recovery research in recent years. The nigrostriatal pathway involves motor planning (Joshua et al. 2009), habit formation (Faure et al. 2005), and reward‐driven motor learning (Widmer et al. 2019; Vassiliadis et al. 2021). Previous studies have shown that lesions directly affecting the basal ganglia increase the risk of poor sensorimotor outcome after stroke (Fries et al. 1993; Boyd and Winstein 2004). One study in well‐recovered stroke patients found that the amount of damage to nigrostriatal white matter tracts, quantified 3 months after stroke, correlated with worse fine motor skills after 1 year (Rimmele et al. 2018). In chronic stroke survivors, the smaller ipsilesional putamen was associated with worse sensorimotor functions (Liew et al. 2021). Animal studies have reported dopamine releases into the ipsilesional striatum shortly after stroke (Globus et al. 1988; Hashimoto et al. 1994), and increased levels of dopamine in the contralesional hemisphere of mice were found to be positively linked to spontaneous recovery (Obi et al. 2018). A supportive role of dopamine for stroke recovery also comes from animal data on brain plasticity and motor skill learning (Rioult‐Pedotti et al. 2015) and traumatic brain injury in which dopaminergic neurotransmission was found to be critically altered after the event, and dopamine levels and dopaminergic interventions have been associated with reductions in oxidative stress and improvement in cellular functions, cell survival, and outcome (Chen et al. 2017; Jenkins et al. 2018).

The present data indicate that patients with more active contralesional SN and PUT at baseline show fewer deficits after stroke, quantified by the NIHSS. This association was evident after correction for the initial deficit and lesion load. Importantly, as group differences between stroke patients and healthy controls were not apparent for ALFF in SN and PUT, their values might be interpreted as a measure of pre‐stroke functional brain reserve or brain capacity in dopaminergic brain circuits to promote functional recovery. Moreover, the data were extracted from the contralesional hemisphere. However, we argue that these data can also be interpreted as a proxy for the ipsilesional hemisphere and their networks involved in recovery processes. In line with this concept of ALFF as an estimate of brain functionality at rest, patients with mild cognitive impairment exhibited decreased ALFF in brain areas critically involved in cognition, including the superior and middle temporal gyrus and inferior parietal lobe (Zhao et al. 2014). However, in terms of connectivity, surprisingly, patients with stronger SN–CAU connectivity seemed to show more impairment at follow‐up. At group level, we did not find a significant upregulation for this connection. Based on the same cohort of patients, we previously reported similar positive associations between more robust couplings of parietofrontal and frontal motor connections and more deficits later after stroke (Backhaus et al. 2021; Braass et al. 2023). In these studies, we have speculated that such connectivity enhancements in contralesional motor networks might be an early but rather unspecific pattern of severely impaired stroke patients at an increased risk for incomplete recovery. The data suggest this might also hold for subcortical brain networks, including the nigrostriatal dopaminergic system.

In the mesocorticolimbic network, we found two opposite effects between interregional coupling and the outcome. On the one hand, a more pronounced VTA–AMYG connectivity was linked to a more significant deficit, whereas a stronger VTA–PFC coupling was correlated with a more favorable outcome. The mesocorticolimbic network is crucial for reward‐related cognitive processes (Lammel et al. 2014; Ruiz‐Tejada et al. 2022). Reward correlates with better motor learning (Abe et al. 2011; Widmer et al. 2016), procedural learning (Wachter et al. 2009), and motor adaptation in healthy patients (Galea et al. 2015). In stroke patients, motor adaptation is also typically impaired. Adding reward or punishment has been shown to improve motor adaptation in stroke patients (Quattrocchi et al. 2017). Aside from many other cognitive functions, the medial PFC has been linked to executive functioning (Deziel et al. 2015). Dopaminergic projections to the PFC have been associated with top‐down executive functions, self‐knowledge, updating goal‐directed behavior, and predicting reward and motivation (Myers et al. 2016; Jobson et al. 2021; Jones and Graff‐Radford 2021). Hence, it would have been intuitive to hypothesize that VTA–AMYG and VTA–PFC connectivity should also be linked to a better outcome. Such a hypothesis would also align with our recent work on the structural reserve of the amygdala and the nucleus accumbens: Larger volumes of these key areas were correlated with a more favorable outcome after a severe stroke (Asmussen et al. 2024). However, as the present data suggests, this is not the case in functional network analyses. “The stronger, the better” might not always hold in dopaminergic networks. Whereas VTA–PFC was positively linked to a better outcome, VTA–AMYG was linked to more impairment. How can we explain this discrepancy? On a first speculative note for VTA–AMYG connectivity, its negative influence could arise from the pathophysiology of post‐stroke depression and mood disorders. In fact, hyperconnectivity between the amygdala, key areas of the default mode network, and salience networks have been related to depressive symptoms in chronic stroke patients (Zhang et al. 2019). In patients with major depressive disorder, studies reported enhanced amygdala responses during emotional and cognitive information processing (Siegle et al. 2007) and stronger FC between VTA and the left amygdala and dorsolateral PFC (Wagner et al. 2017). Unfortunately, data regarding premorbid depressive symptoms and post‐stroke depression were not available for the present cohort of patients. Future prospective analyses are needed to systematically explore to what extent baseline VTA–AMYG in stroke patients might show a correlation with the risk of post‐stroke depression and to validate the negative impact of this coupling measure for overall outcome after stroke. In contrast to VTA–AMYG, we found a positive link between baseline VTA–PFC FC and a better outcome after stroke; patients with stronger coupling between VTA and the PFC would have a better chance for a better outcome. This result would find support from some functional studies, which have revealed that, among many other regions, prefrontal non‐motor areas are mainly activated in motor tasks in more severely impaired patients and can also inform about future recovery (Ward et al. 2003a, 2003b). In agreement, few connectivity studies in chronic stroke patients found enhanced couplings between cortical sensorimotor areas and prefrontal cortices, positively related to better motor functions (Sharma et al. 2009; Varkuti et al. 2013; Chu et al. 2023), interpreted as the brain's attempt to activate available resources for functional compensation.

Apart from these significant associations, we also found that some areas, such as the caudate nucleus, VTA, the nucleus accumbens, the hippocampus, and the PFC, exhibited increased ALFF but did not correlate with the clinical scores. We argue that this will likely be an unspecific pattern of brain activation and the brain's attempt to recruit resources to compensate for the lost functions. An upregulation of ALFF of the motor cortex has been reported in subacute subcortical stroke patients (Liu et al. 2015). Such observations also align with numerous task‐based fMRI studies (Sharma et al. 2009; Rehme et al. 2012; Favre et al. 2014) showing widespread upregulations not only in motor‐related brain networks but also in cognition‐related networks (Marshall et al. 2009; Li et al. 2016). These were explained by attentional processes to motor performance (Ward et al. 2003a) or by motor learning strategies (Buma et al. 2016), particularly early after stroke and in patients with more severe motor deficits. However, in many cases, they were not linked with clinical scores or recovery trajectories.

Based on our findings, it is worth considering whether dopaminergic therapy might alter ALFF and FC within the dopaminergic network and thereby influence clinical outcomes in stroke patients. In the Enhancement of STroke REhabilitation with Levodopa (ESTREL) study, stroke patients received levodopa therapy in addition to standardized rehabilitation therapy. Although the results of the ESTREL trial (Engelter et al. 2025) showed no significant effect of the additional levodopa therapy alongside standardized rehabilitation therapy, the findings cannot be generalized to the entire group of stroke patients. The authors of the ESTREL study themselves, as well as a commentary in JAMA (Lin 2026), relativize their findings by pointing out that the results do not prove that levodopa therapy has no potential effect whatsoever on improving motor function following a stroke, as genetic factors and individual differences in levodopa metabolism may mean that levodopa therapy needs to be individually tailored to achieve a significant effect. Further studies are needed to investigate the effect of dopaminergic therapy on stroke rehabilitation in greater depth.

There are several significant limitations to note. First, this explorative analysis is based on only a small sample of acute stroke patients exhibiting severe initial impairment. Although results were corrected for multiple comparisons, sensitivity and specificity are limited, and validation in prospective future studies is crucial to verify or falsify the present findings. Second, in line with our previous work on the structural reserve of dopaminergic key areas, the whole extent of the regions of interest was considered in the analyses. A subcomponent analysis, for example, parcellating the amygdala into its centromedial or laterobasal parts, was not in the scope of this work (Ambrosi et al. 2017). Also, the AAL3 atlas was chosen for region selection in agreement with our previous report (Asmussen et al. 2024). Based on 7‐T MRI data, advanced atlases for subcortical nuclei might further improve their delineation in standard space and alter the present results (Bianciardi et al. 2015; Mohammadi et al. 2024). Third, in this study, relevant cognitive and psychiatric impairment, including major depression, was an exclusion criterion. As most patients had severe deficits, a detailed cognitive/psychiatric assessment was neither included nor would it have been feasible at the initial time point. To what extent the premorbid cognitive/psychiatric conditions or the development of post‐stroke depression might have biased the present group differences and ALFF/FC‐outcome associations remains unclear. Fourth, we did not systematically collect information regarding the type and amount of rehabilitation or family support between time points T1 and T2. In the future, larger samples, including such additional information, might alter the present outcome associations. Fifth, although similar acquisition durations of resting‐state functional MRI sequences have been used in other studies (Martino et al. 2018), a longer acquisition duration would be desirable but is not feasible, especially in this group of severely affected stroke patients. Sixth, because some of the ROIs studied are very small (e.g., SN), a smaller voxel size might be better, but resting‐state MRI sequences with larger voxels have been successfully used to study these regions (Martino et al. 2018; Zang et al. 2022). Seventh, because a group of older patients was examined, preexisting white matter lesions may have influenced FC. Because evaluable T2‐weighted data were not available for all patients and control subjects, this factor was not included in the models. Nevertheless, the available Fazekas scores were included in Table 1. Eighthly, the paper examined dopaminergic regions. However, as no other data are available, such as information on dopaminergic function obtained via DAT‐SPECT, it is theoretically possible that non‐dopaminergic influences also play a role. Finally, in line with our previous work (Rojas Albert et al. 2022; Braass et al. 2023; Asmussen et al. 2024), we focused our analyses on the unaffected hemisphere to exclude any direct lesion effects. The correlation between ALFF or FC measures and clinical outcomes may therefore differ across dopaminergic ipsilesional regions. Future studies are warranted to explore how baseline activation and coupling data in dopaminergic networks, spared from the primary stroke lesion, would associate with recovery trajectories after stroke.

## Author Contributions

**Hanna Braaß:** formal analysis, methodology, writing – original draft, writing – review and editing. **Liv Asmussen:** methodology, visualization, writing – review and editing. **Winifried Backhaus:** investigation, methodology, writing – review and editing. **Focko L. Higgen:** methodology, writing – review and editing. **Philipp J. Koch:** methodology, writing – review and editing. **Pawel Wrobel:** methodology, writing – review and editing. **Chi‐un Choe:** methodology, writing – review and editing. **Fanny Quandt:** methodology, writing – review and editing. **Benedikt Frey:** methodology, writing – review and editing. **Robert Schulz:** conceptualization, formal analysis, methodology, writing – original draft, writing – review and editing.

## Funding

This work was funded by the Else Kröner‐Fresenius‐Stiftung (2016_A214 to R.S.). R.S. and C.C. are supported by an Else Kröner Exzellenzstipendium from the Else Kröner‐Fresenius‐Stiftung (2020_EKES.16 to R.S. and 2018_EKES.04 to C.C.). F.Q. is supported by the Gemeinnützige Hertie‐Stiftung (Hertie Network of Excellence in Clinical Neuroscience). F.L.H. and P.W. are supported by the German Research Foundation (DFG) and the National Science Foundation of China (NSFC) in the project Crossmodal Learning, SFB TRR 169/A3.

## Conflicts of Interest

The authors declare no conflicts of interest.

## Data Availability

Due to restrictions given by the Ethics Committee, the data may be shared only upon reasonable request, but not in a publicly available repository.
